# Literary Notes

**Published:** 1907-03

**Authors:** 


### LITERARY NOTES

The Annals of Otology, Rhinology and Laryngology for December, 1906, edited by Dr. H. W. Loeb, Saint Louis, forms a Fraenkel Festschrift number. It is a handsome edition containing ninety-four titles, well illustrated and embellished with a frontispiece portrait of Professor Fraenkel.

---

The Mississippi Valley Medical Association offers a prize of $100 for the best essay on a medical or surgical subject written by a member of the association. All essays must be typewritten, and are to be sent to the Secretary, Dr. Henry Enos Tuley, 111 W. Kentucky Street, Louisville, on or before August 1, 1907.

